# Management of Persistent Thumb-Sucking Habit Using a Modified Thumb Guard Appliance: A Case Report

**DOI:** 10.7759/cureus.111565

**Published:** 2026-06-26

**Authors:** Sandisha S Sudrik, N D Shashikiran, Namrata Gaonkar, Rutuja B Salunkhe, Ruchira R Sawant

**Affiliations:** 1 Department of Paediatric and Preventive Dentistry, School of Dental Sciences, Krishna Vishwa Vidyapeeth, Karad, IND

**Keywords:** digit-sucking habit, habit-breaking appliance, interceptive treatment, pediatric dentistry, thumb guard, thumb sucking

## Abstract

Thumb sucking is a common oral habit during early childhood and is generally considered normal during infancy. However, persistence of the habit beyond the preschool years may result in adverse dentofacial changes, including increased overjet, anterior open bite, and altered oral function. Early intervention is often required to prevent the progression of these malocclusions. A 5-year-old boy presented with a persistent thumb-sucking habit of one year's duration, predominantly during daytime hours. Clinical examination revealed a high-arched palate and the presence of a callus on the thumb, consistent with chronic digit-sucking behavior. Considering the child's age and the parents' preference to avoid a fixed habit-breaking appliance, a modified removable thumb guard appliance was designed and fabricated. The appliance was delivered along with appropriate counseling and positive reinforcement strategies to facilitate habit cessation. The child demonstrated good acceptance and compliance with the appliance throughout the follow-up period of six months. A gradual reduction in the frequency of thumb sucking was observed, with complete cessation of the habit achieved within three months of appliance use. The callus on the thumb showed progressive regression and had significantly resolved by the end of the follow-up period. No further episodes of thumb sucking were reported by the parents. The appliance was well tolerated, and no complications or adverse effects were observed. Owing to the relatively short follow-up duration, no appreciable changes in the high-arched palate were noted. In the present case, the modified thumb guard appliance was associated with successful cessation of the thumb-sucking habit and was well accepted by the child throughout the treatment period. This appliance may be considered a non-invasive and patient-friendly option for managing persistent thumb-sucking habits in young children. This approach may be considered a viable alternative when conventional fixed habit-breaking appliances are not preferred by parents or caregivers. However, further studies with larger sample sizes are required to evaluate its long-term efficacy and broader clinical applicability.

## Introduction

Oral habits are considered a normal part of childhood development, often emerging as learned patterns of muscle contraction with complex behavioral components. Among these, sucking habits are the most common, typically beginning in utero around the 29th week of gestation and continuing into infancy as a reflexive behavior that provides comfort and security [1,2]. Infants and young children frequently engage in thumb, finger, or pacifier sucking, particularly during periods of anxiety, separation, or when seeking relaxation before sleep [3]. While such habits are generally self‑limiting and resolve by two to four years of age, persistence beyond this period can adversely affect the developing dentition and occlusion [1].

Prolonged thumb sucking has been associated with anterior open bite, increased overjet, lingual inclination of mandibular incisors, labial proclination of maxillary incisors, posterior crossbite, and speech disturbances [2-4]. The severity of these changes depends on the duration, frequency, and intensity of the habit, with children engaging in prolonged sucking during the eruption phase of permanent teeth being particularly susceptible to dentoalveolar and skeletal abnormalities [3,4]. The prevalence of thumb-sucking habits is the highest during infancy and early childhood, with studies reporting rates of approximately 70-90% in newborns and infants, decreasing to 15-30% among preschool children and less than 5-10% in children older than 8 years. Persistence of the habit beyond the preschool years has been associated with several factors, including parental education, feeding practices, psychosocial stress, and behavioral and environmental influences [5-7].

Removable habit-breaking appliances, including conventional thumb guards and oral screens, are widely used because they are relatively simple, non-invasive, and economical. However, their success largely depends on patient motivation and compliance, and they may not provide the positive reinforcement and distraction associated with newer habit-interception approaches. In contrast, the Bluegrass appliance employs a non-punitive roller mechanism that redirects the child's attention and promotes neuromuscular stimulation, resulting in improved acceptance and compliance. To the best of our knowledge, there are limited reports describing the incorporation of a Bluegrass-type roller into a removable thumb guard appliance. The present modification was designed to combine the barrier function of a thumb guard with the distraction-based mechanism of the Bluegrass appliance, providing a removable, child-friendly option for managing persistent thumb-sucking habits in young children [8-9]. In 1991, Haskell and Mink introduced the Bluegrass appliance, a non‑punitive, distraction‑based device incorporating a roller that encourages neuromuscular stimulation and habit reversal [10-14]. Subsequent modifications, including the Lingual Pearl and Baker’s multi‑roller designs, have expanded its clinical applications across primary and permanent dentitions [15,16].

This case report presents the management of a 5‑year‑old child with persistent thumb sucking, where parental reluctance toward fixed intraoral appliances necessitated an alternative approach. A customized thumb guard was fabricated and modified by incorporating a Bluegrass‑inspired roller element. This modified appliance combined a physical barrier with a distraction mechanism, offering a non-invasive and parent-accepted approach for managing persistent thumb-sucking habits. The present case highlights the importance of individualized treatment planning and appliance modification tailored to the child's needs and parental preferences in pediatric habit interception.

## Case presentation

**Figure 1:**
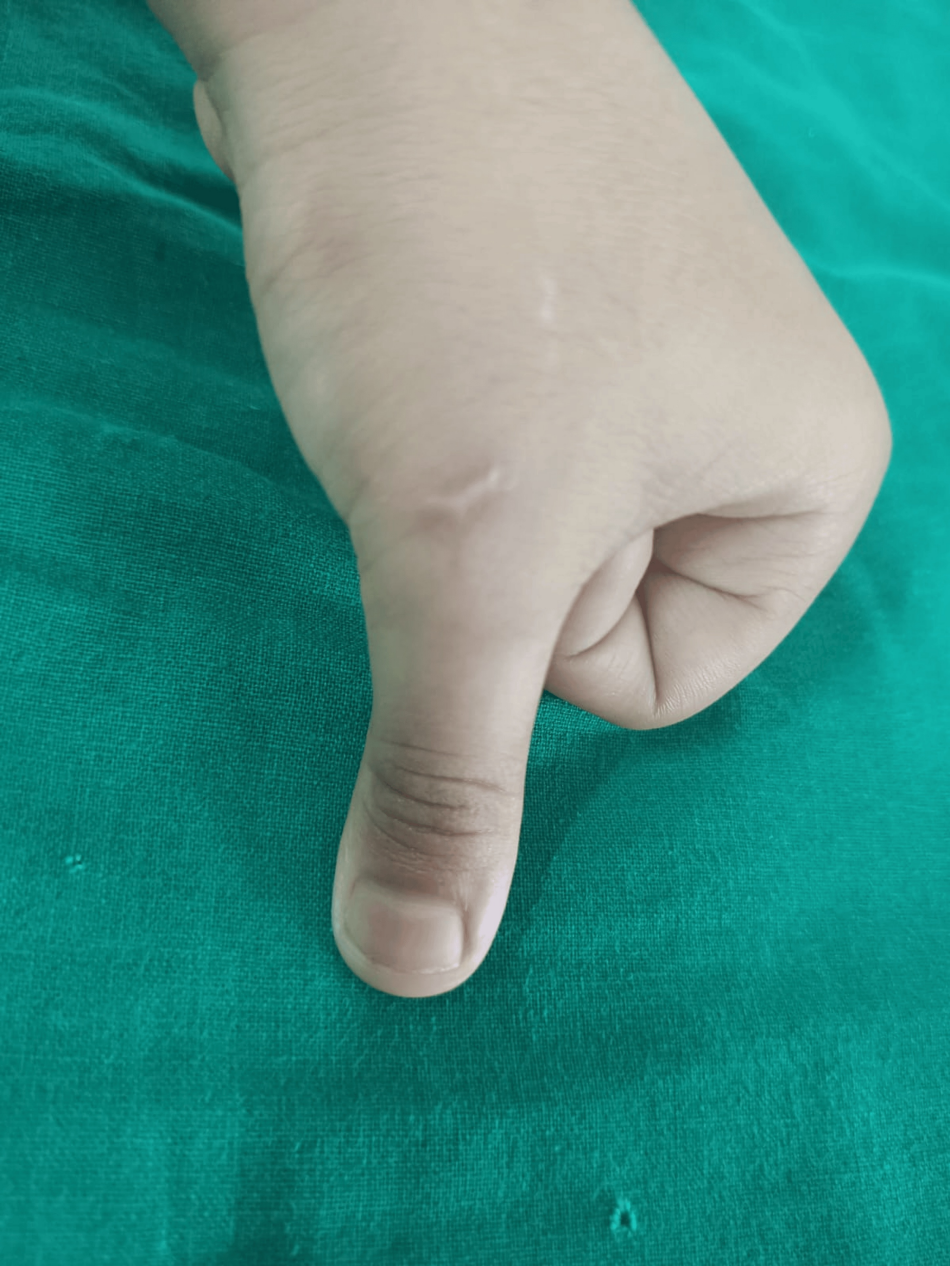
Clinical image of the patient’s thumb showing callus formation due to a prolonged thumb-sucking habit.

A 5-year-old boy was brought by his parents with a persistent thumb-sucking habit since early childhood. The parents reported that the child engaged in thumb sucking unconsciously during idle periods, averaging seven to eight hours per day. The child was medically healthy, with no history of systemic illness, developmental disorders, or psychological concerns. Prenatal, natal, and postnatal histories were non-contributory, and developmental milestones had been achieved appropriately for age. Dental history revealed no previous habit-interception or orthodontic treatment. The child belonged to a supportive family environment, and no significant psychosocial stressors or behavioral issues associated with the persistence of the habit were identified. Clinical examination revealed a high palatal vault and callous formation on the thumb, consistent with chronic thumb-sucking behavior. No other significant dentofacial abnormalities, such as anterior open bite, increased overjet, posterior crossbite, or facial asymmetry, were observed at the time of examination. As this was an early interceptive case and objective measurements of palatal morphology were not recorded, qualitative clinical assessment was used to document the habit-related changes (Figure 1). The parents expressed reluctance toward fixed intraoral habit‑breaking appliances, necessitating an alternative removable approach.

At the initial visit, a comprehensive history of the thumb-sucking habit was obtained, including the age of onset, duration, frequency, and circumstances associated with the habit. A clinical examination was performed to assess habit-related oral and dentofacial changes, which revealed a high palatal vault and callous formation on the thumb. The patient's medical, dental, developmental, and psychosocial histories were also reviewed and found to be non-contributory. Following the diagnostic assessment, counseling was provided to both the child and parents regarding the ill effects of prolonged thumb sucking on the developing dentition and the available treatment options. Mirror Dunlop’s technique was employed to enhance motivation, wherein the child was asked to observe his habit in the mirror and reflect on whether he truly wished to discontinue it. The child expressed willingness to stop the habit, which strengthened compliance.

**Figure 2:**
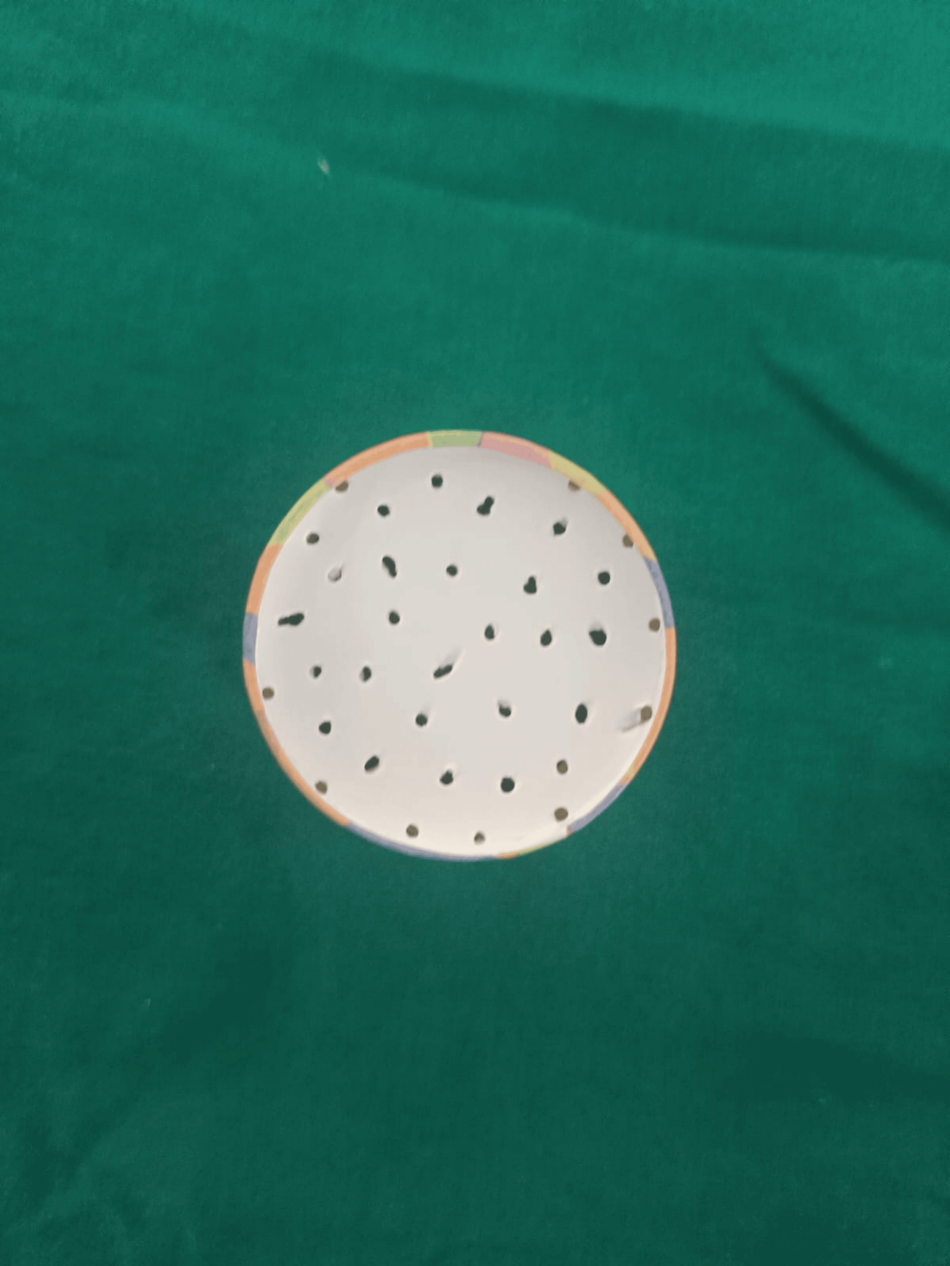
Plastic cup with holes at the base used for alginate thumb impression.

**Figure 3:**
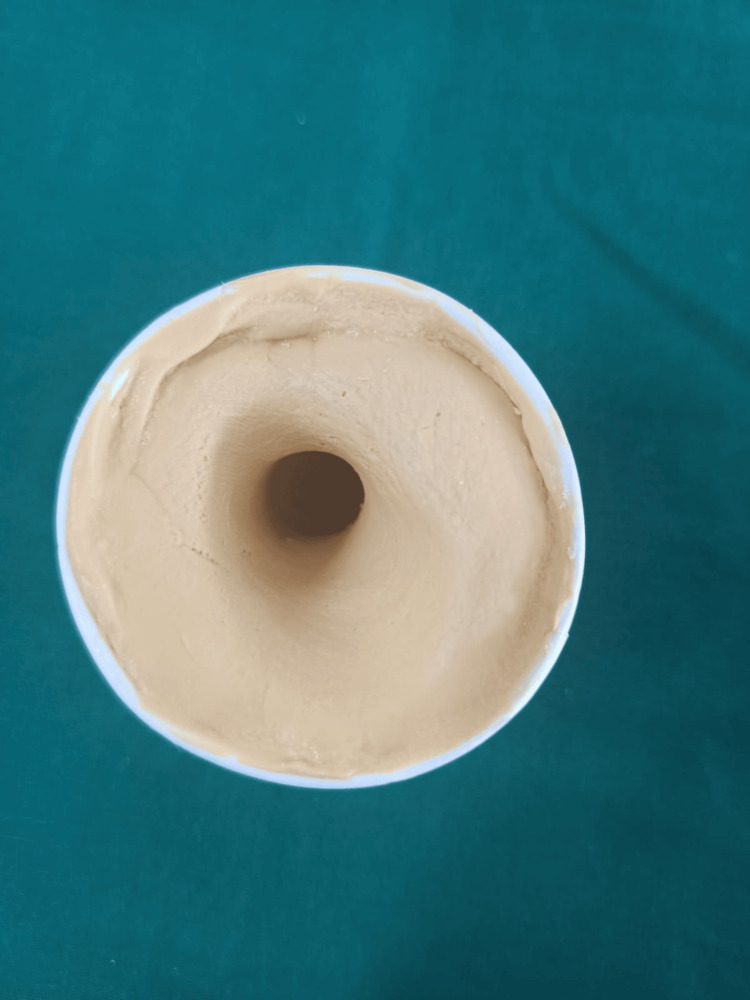
Alginate thumb impression in a plastic cup

A removable thumb guard with a novel modification was planned. For appliance fabrication, a modified impression technique was used to improve patient comfort and facilitate accurate recording of the thumb for appliance fabrication. A plastic cup was modified by creating multiple holes at its base to allow excess alginate material to escape (Figure 2). Alginate impression material was placed into the cup, and the child was instructed to insert his thumb upside down into the cup to obtain an accurate impression (Figure 3). The impression was then poured with dental stone to create a working cast.

**Figure 4:**
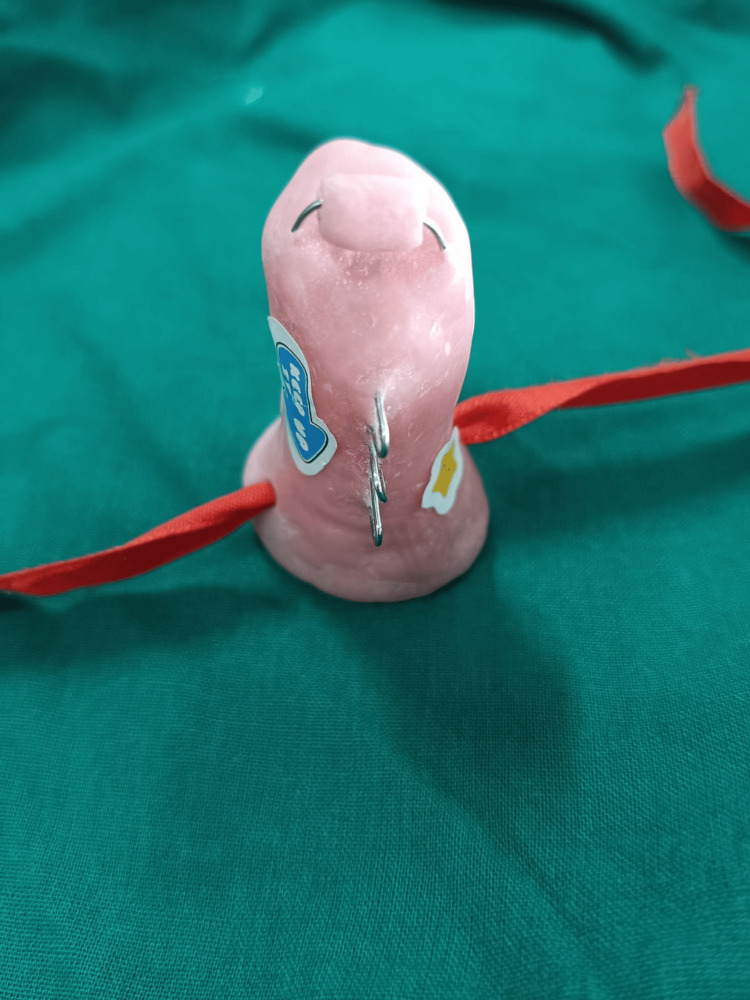
Removable thumb guard with Bluegrass roller incorporated at the tip

A removable thumb guard was fabricated using cold‑cure acrylic resin. Stainless steel wire components (19‑gauge) were adapted to provide structural reinforcement (Figure 4). A Bluegrass‑inspired roller was fabricated using wire and acrylic and attached to the guard. This roller element acted as a distraction mechanism, encouraging the child to manipulate the roller instead of engaging in thumb sucking. At the base of the guard, two opposite holes were created to allow a ribbon to pass through, enabling the appliance to be securely tied to the thumb.

The appliance was delivered and fitted comfortably. Instructions were given to the child to roll the Bluegrass element whenever the urge to suck the thumb occurred. Monthly follow‑ups were scheduled to monitor compliance and appliance integrity. The child adapted well to the appliance, actively engaging with the roller, and demonstrated progressive reduction in thumb-sucking behavior. By the end of two months, callous formation on the thumb had resolved, and the habit was discontinued. The appliance was removed after complete cessation of the thumb-sucking habit. The patient was monitored for six months following appliance removal, and no signs of relapse were observed during this period.

This case describes the use of a modified removable thumb guard incorporating a Bluegrass roller, combined with motivational counseling, for the management of a persistent thumb-sucking habit in a young child. Complete cessation of the habit was achieved, accompanied by significant resolution of the thumb callus, and no relapse or appliance-related adverse events were observed during the follow-up period. The present case suggests that this modified appliance may be a non-invasive and patient-friendly option for selected pediatric patients; however, further studies are required to evaluate its long-term effectiveness and broader clinical applicability.

## Discussion

Digit sucking is one of the earliest forms of habitual manipulation observed in children and, if prolonged, can adversely affect the developing dentition and occlusion [1,2]. The persistence of such habits beyond the preschool years has been associated with malocclusion, high palatal vault, anterior open bite, increased overjet, and posterior crossbite [8-12]. The timing of intervention remains a critical question for clinicians, as early cessation of the habit reduces the likelihood of skeletal and dentoalveolar changes [17,18].

Traditional habit‑breaking appliances, such as palatal cribs, spurs, hay rakes, and cage‑type devices, have been employed for decades [19]. While effective in some cases, these appliances often rely on aversive stimuli and physical barriers, which may cause emotional disturbances, speech difficulties, eating problems, or even self‑inflicted trauma. Furthermore, appliances such as hay rakes and cages are prone to damage during mastication or habitual manipulation, and children may perceive them as punitive measures [19]. This highlights the need for more child‑friendly, non‑punitive alternatives.

In the present case, the child exhibited a persistent thumb-sucking habit associated with a high palatal vault and callous formation on the thumb, indicating chronic habit persistence. Considering the patient's young age and the parents' preference to avoid a fixed intraoral appliance, a modified removable thumb guard incorporating a Bluegrass roller was selected. The appliance was well accepted by the child and, in conjunction with motivational counseling, was associated with gradual reduction and eventual cessation of the habit. The thumb callus resolved completely during follow-up, and no appliance-related complications or relapse were observed.

Previous studies on the Bluegrass appliance have reported high patient acceptance and successful habit cessation, with the duration of treatment varying according to the child's age, motivation, and severity of the habit [14-16]. Younger children are generally reported to adapt more readily and achieve habit cessation earlier than older children [17,18]. The favorable outcome observed in the present case is consistent with these reports and suggests that combining the barrier effect of a removable thumb guard with the distraction-based mechanism of the Bluegrass roller may offer a practical alternative for selected pediatric patients. Nevertheless, as this report describes a single case with qualitative clinical outcomes, further studies with larger sample sizes and objective outcome measures are needed to evaluate the long-term effectiveness of this modified design [20].

In the present case, parental reluctance toward intraoral appliances necessitated an innovative extraoral solution. A removable thumb guard was fabricated using cold‑cure acrylic resin, reinforced with 19‑gauge wire, and modified by incorporating a Bluegrass‑inspired roller. The impression was obtained using a simple plastic cup technique, with escape holes at the base to allow excess alginate flow, ensuring accuracy and comfort. To secure the appliance, two opposite holes were created at the base of the guard, through which a ribbon was passed and tied around the thumb. This design provided both a physical barrier and a distraction mechanism, encouraging the child to manipulate the roller instead of sucking the thumb. Importantly, Mirror Dunlop’s motivational technique was employed before appliance delivery, ensuring the child’s willingness to discontinue the habit.

In the present case, complete cessation of the thumb-sucking habit was achieved within six months, with no relapse observed during the follow-up period. However, this outcome was likely the result of multiple interacting factors, including motivational counseling, positive reinforcement, active parental involvement, and the use of the modified removable thumb guard incorporating a Bluegrass roller. The appliance was well accepted by both the child and parents and was associated with good compliance throughout treatment. Compared with conventional intraoral habit-breaking appliances, the present design was non-punitive, esthetic, and easy to wear, which may have contributed to its acceptability in this case. Nevertheless, as this is a single case report, no definitive conclusions regarding the superiority or effectiveness of the appliance can be drawn, and further studies are required to evaluate its clinical performance. The distraction‑based mechanism minimized psychological stress, while the removable nature of the appliance avoided complications related to speech, mastication, or tissue irritation. Thus, this case suggests that a modified thumb guard incorporating a Bluegrass roller may represent a practical and parent-accepted option for habit interception in selected young children, although further studies are needed to evaluate its long-term effectiveness and broader clinical applicability.

## Conclusions

The present case demonstrates that a removable thumb guard incorporating a Bluegrass roller can serve as an effective, child‑friendly alternative for intercepting persistent digit sucking habits. Fabricated using a simple plastic cup impression technique and secured with ribbon, the appliance was well accepted by both the child and parents. The distraction‑based mechanism redirected the child’s neuromuscular activity toward playing with the roller, resulting in cessation of the habit within two months and resolution of callous formation, without relapse during follow‑up.

The present removable design was well accepted by both the child and parents and was associated with good compliance throughout the treatment period. No appliance-related adverse events were observed during follow-up. When combined with motivational counseling techniques, such as Mirror Dunlop’s method, the appliance provided a holistic approach to habit correction. Thus, this modified removable appliance may be considered a practical and esthetic option for habit interception in selected young children. In the present case, it was well accepted by the child and parents and was associated with good compliance and successful habit cessation. However, larger studies are needed to confirm these findings and assess long-term outcomes.
